# Does Patient Weight, Age, or Gender Correlate With the Ability to Visualize the Distal Aorta on Bedside Aortic Ultrasounds in the Emergency Department?

**DOI:** 10.7759/cureus.33822

**Published:** 2023-01-16

**Authors:** Christopher Bryczkowski, Bhavesh Mody, Grant Wei, Jonathan V Mccoy, Rajesh Geria, Mary Rometti

**Affiliations:** 1 Emergency Medicine, Rutgers Robert Wood Johnson Medical School, New Brunswick, USA

**Keywords:** emergency ultrasound, emergency medicine ultrasound, aortic ultrasound, abdominal aorta, abdominal obesity, pocus (point of care ultrasound)

## Abstract

Introduction: Abdominal aortic aneurysms (AAA) have a varied presentation, which often makes the diagnosis difficult. The most common location for an AAA is in the infra-renal or distal aorta, which can be difficult to visualize using bedside ultrasound.

Objective: This study was designed to identify if a patient’s weight, gender, or age influenced our ability to visualize the distal aorta on bedside abdominal aortic ultrasound scans.

Methods: All aortic scans completed in the Emergency Department (ED) from September 2010 to September 2013 were retrospectively evaluated. Patients 21 years and older were included. Scans missing age, gender, or self-reported weight were excluded.

Results: 500 aortic scans were included. The distal aorta was visualized in 393 scans (78.6%). The mid aorta was visualized in 417 scans (83.4%). The proximal aorta was visualized in 454 scans (90.8%). For the distal aorta, the average weight for visualized versus not visualized was 75.7 kg versus 79.7 kg. For the proximal aorta, the average weight for visualized versus not visualized was 75.8 kg versus 84.0 kg. Weight significantly predicted the ability to visualize the proximal aorta (unadjusted p=0.0098, adjusted p=0.0095) and marginally predicted the ability to visualize the distal aorta (unadjusted p=0.071, adjusted p=0.019). Neither age (unadjusted p=0.13, adjusted p=0.052) nor gender (unadjusted p=0.74, adjusted p=0.40) was significantly associated with visualization.

Conclusion: There is no clinically significant difference in the ability to visualize a patient’s distal aorta with bedside ultrasound based on a patient’s body weight, gender, or age.

## Introduction

Developing an abdominal aortic aneurysm (AAA) is not uncommon, with the lifetime risk of being diagnosed with an AAA about 1 in 17 for the general population and about 1 in 9 for current smokers [1,2]. The patient presentation of a AAA can vastly vary, making it a difficult diagnosis [3,4]. Few patients with AAA or ruptured AAA present with the classic triad of abdominal pain, hypotension, and pulsatile mass [1,4-9]. Therefore, the diagnosis of a AAA may be missed or delayed, with potentially disastrous results [3,4,10-12]. Moreover, nearly 33% of patients do not know that they have an AAA [13], with one source citing over 80% of AAA lacking a previous diagnosis when the aortic rupture occurred [3]. Ruptured AAAs have a mortality rate close to 90% [3,4,7,14,15].

Ultrasound has been used for many years as an adjunct to the diagnosis of AAA, especially as an outpatient screening exam [1,5]. Additionally, in patients with a known AAA, ultrasound is often used to monitor the size progression of the aneurysm [5,14]. For symptomatic patients in the emergency department (ED), the bedside ultrasound has become an essential tool in establishing or excluding the diagnosis of a AAA [1,3,6,15,16]. In an undifferentiated patient, bedside ultrasound is especially useful to begin searching for the diagnosis in a critically ill, hemodynamically unstable patient. Although computed tomography (CT) is still required to determine if the AAA is leaking, the presence of symptoms and dilated aorta on ultrasound is typically enough to suggest a positive diagnosis and expedite surgical consultation and treatment [4].

While use is operator-dependent, ED physicians with limited AAA ultrasound training can identify AAA in patients with nearly 100% accuracy [17]. Bedside ultrasound is almost 100% sensitive and specific for diagnosing AAAs [6,7]. Furthermore, bedside ultrasound was shown to be comparable to conventional radiology ultrasound for the diagnosis of AAA [18]. Internal medicine residents who completed four one-on-one sessions on the use of ultrasound (US) to detect AAA were able to identify AAA in patients with nearly the same accuracy as a credentialed sonographic instructor [19].

Patients of different genders, races, and body habitus undergo bedside AAA ultrasound exams in the ED. The most common location for an AAA is the infra-renal (distal) aorta. While bedside ultrasound offers a unique way to assess for AAA, patient characteristics may limit the ability to obtain a meaningful scan [6]. Because of various physical attributes (body habitus, bowel gas, scars), the distal aorta can be especially difficult to visualize. In one radiology study, nearly 33% of inconclusive abdominal ultrasounds demonstrated pathology in follow-up CT scans [20]. Our primary goal was to identify if a patient’s weight influenced the ability to visualize the aorta on bedside abdominal aortic ultrasound scans. Our secondary goal was to determine if a patient’s gender influenced our ability to visualize the aorta on bedside aortic ultrasounds.

This article was previously presented at the American College of Emergency Physicians Scientific Assembly Research Forum 2014.

## Materials and methods

**Figure 1:**
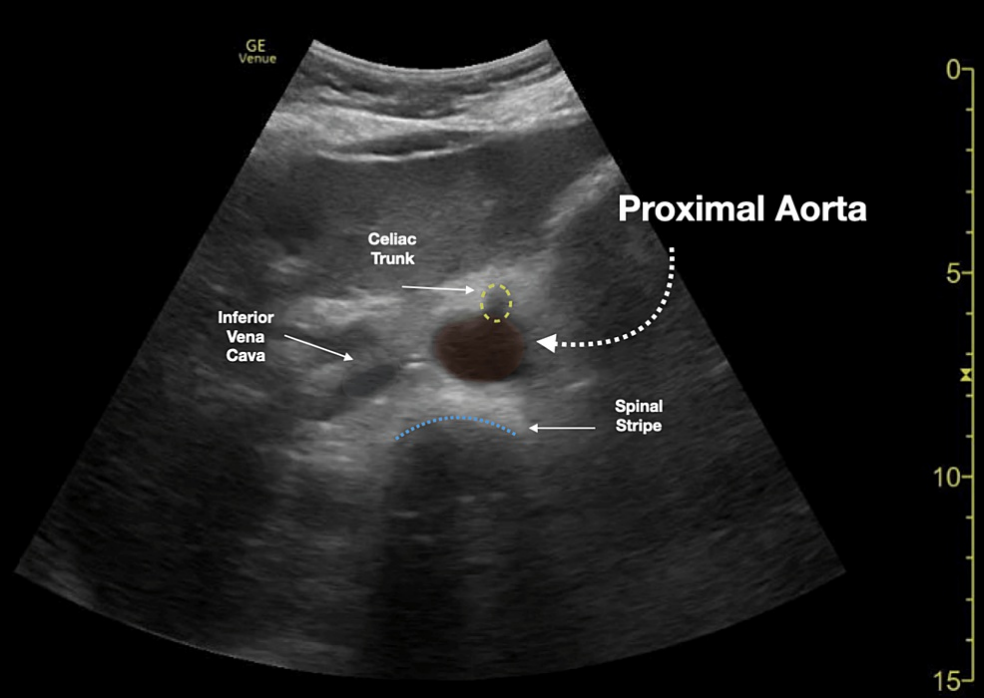
Proximal aorta ultrasound

**Figure 2:**
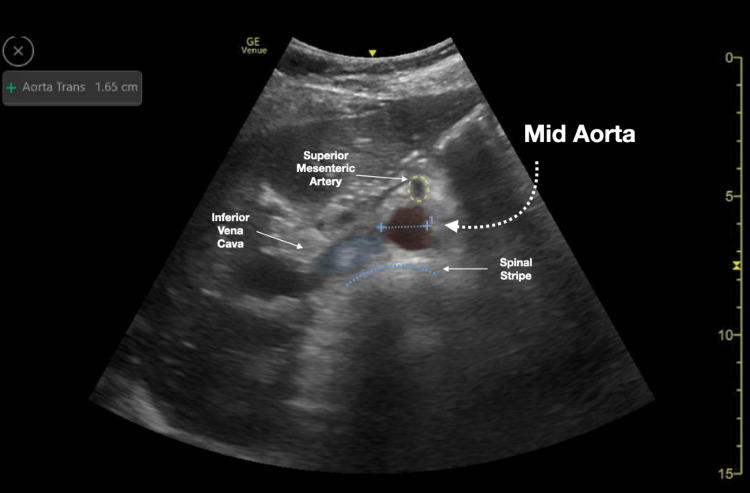
Mid aorta ultrasound

**Figure 3:**
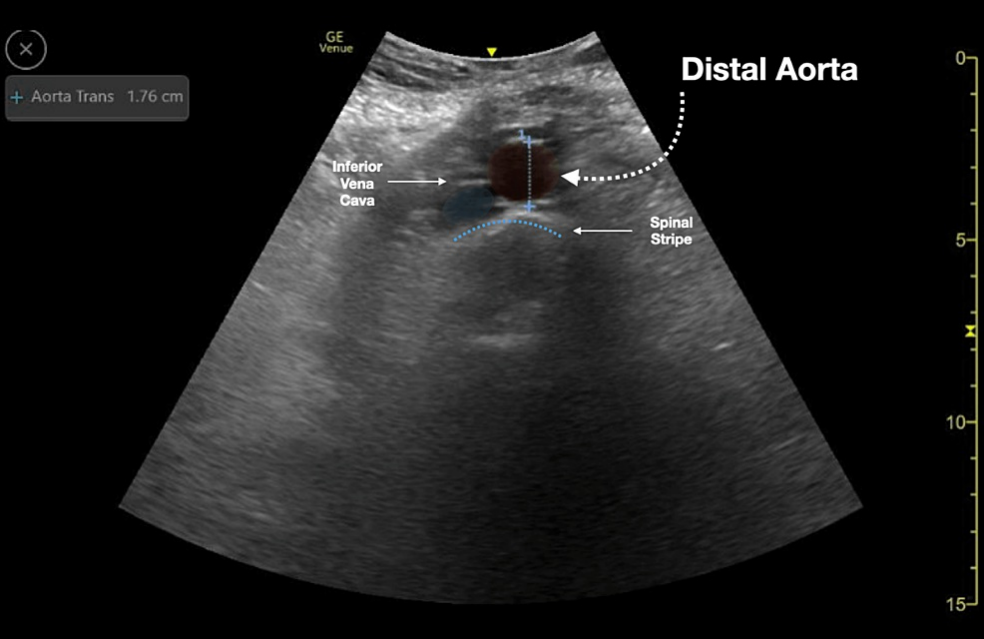
Distal aorta ultrasound

**Figure 4:**
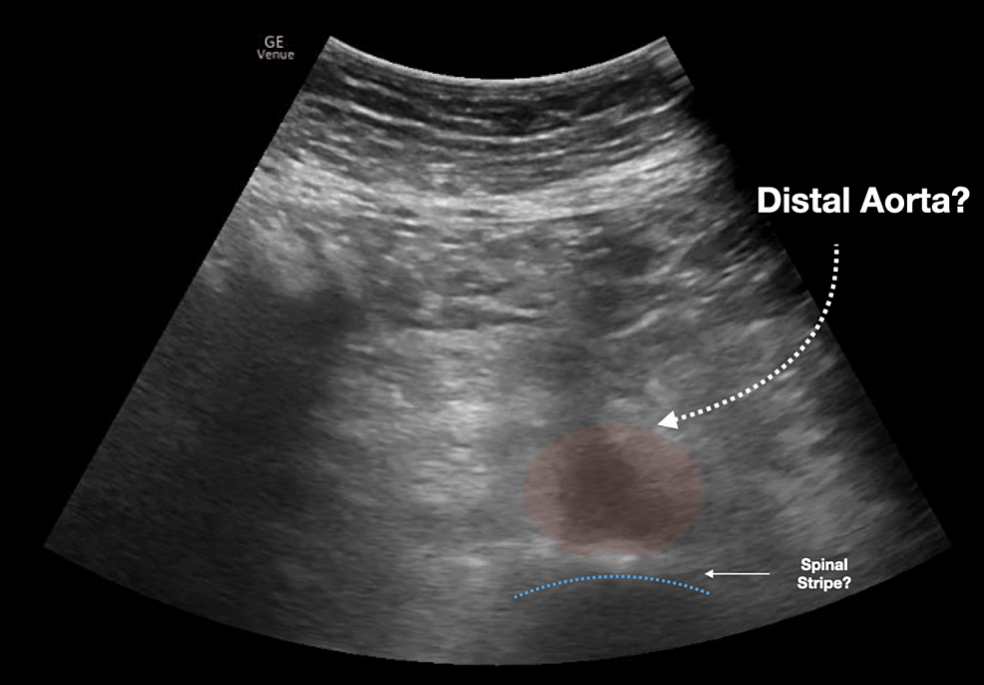
Difficult to visualize the distal aorta In this ultrasound image, shadowing and other artifacts make it difficult to visualize the distal aorta.

We conducted a retrospective chart review of emergency department (ED) bedside aortic ultrasound scans completed at an academic tertiary urban level one trauma center. All aortic scans were completed in the emergency department under the supervision of qualified ED physicians and were recorded/stored in a quality control database (Qpath © 2011 Telexy Healthcare, Inc.). Subsequently, a worksheet reviewing the scan and providing an interpretation was completed by the person performing the bedside ultrasound. The worksheet and accompanying ultrasound images were reviewed by an emergency ultrasound fellowship-trained physician to ensure the adequacy of scan interpretations. Though measurements of the aorta could be taken at multiple areas along the aorta, this study focused on whether the proximal, mid, and distal aorta was adequately visualized. Figures 1-3 demonstrate sample ultrasound images of the proximal, mid, and distal aorta. In these images, the aorta and surrounding structures are easily identified and measured. On the other hand, Figure 4 shows an example of an ultrasound image of the distal aorta, which is difficult to clearly distinguish from surrounding structures.

A list of aortic scans between September 2010 and September 2013 was extracted from the database, and the age, gender, and self-reported weight were extracted from the electronic medical record by an unblinded reviewer. We included all patients 21 years and older who presented to the emergency department during the study period and had a bedside abdominal aortic scan completed and reviewed in Qpath. Patients below the age of 21, whose gender was unknown, or whose self-reported weight was not recorded were excluded. Incomplete Qpath worksheets were also excluded. All included aortic scans were already being completed for routine clinical purposes. Scans were completed using a Zonare (Z.One Ultra, Mountain View, CA) or Sonosite (M-Turbo, Bothell, Washington) device. All ED physicians had training on the operation of these ultrasound devices previously, enabling them to be familiar with their functions. The medical school’s Institutional Review Board approved this study.

The means, standard deviations, and histograms were used to summarize the distributions of age and weight, while frequencies summarized the distributions of gender and visualization of aortas. Logistic regression was used to assess the adjusted and unadjusted effects of weight, gender, and age on visualization of the proximal, mid, and distal aorta.

## Results

A total of 711 aortic scans were completed and recorded in the database during the study period. Of the 711 completed scans, 185 were excluded from the study because of incomplete worksheets. An additional 26 scans were excluded because age, gender, and weight were inappropriately documented or missing. A total of 500 aortic scans were included. The average patient age was 67 ± 19 years. The average weight was 76.6 ± 20.1 kg. 264 males and 236 females were included in the study (Table 1). The distal aorta was adequately visualized in 393 scans (78.6%). The mid aorta was adequately visualized in 417 scans (83.4%). The proximal aorta was adequately visualized in 454 scans (90.8%). The rate of visualization was greatest for the proximal aorta, followed by the mid aorta, and lastly by the distal aorta (Table 2). The most common reason cited for the non-visualization of any part of the aorta was the presence of bowel gas. Other reasons for the non-visualization of the aorta were patient noncompliance and equipment malfunction. The average weight of patients in whom the distal aorta was adequately visualized was 75.7 kg, while the average weight of patients in whom the distal aorta was not adequately visualized was 79.7 kg (unadjusted p=0.071, adjusted 0.019). The average weight in males for whom the distal aorta was adequately visualized and not adequately visualized was 81.0 kg and 84.9 kg, respectively. The average weight in females for whom the distal aorta was adequately visualized and not adequately visualized was 69.7 kg and 74.1 kg, respectively. Weight significantly predicted visualization for the proximal aorta (unadjusted p=0.0098, adjusted p=0.0095) and was marginally associated with visualization of the distal aorta (unadjusted p=0.071, adjusted p=0.019). Neither age nor gender was significantly associated with the visualizations (Table 3). Additional analyses found that the effects of weight or age on visualization did not differ significantly by gender (all p>0.20).

**Table 1:** Means and standard deviations (age, weight) or numbers and percentages (male gender) for patients included and excluded from the analysis.

Demographics	Included (N=500) Mean (SD) or N (%)	Excluded (N=170) Mean (SD) or N (%)	P-value
Age (Years)	67.0 (18.5)	58.1 (20.1)	<0.0001
Weight (Kg)	76.6 (19.8)	80.5 (21.9)	0.029
Gender (Male)	264 (52.8%)	86 (50.6%)	0.62

**Table 2:** Rates and percentages of aortic visualization

Visualization of	Included (N=500) N (%)
Proximal Aorta	454 (90.8%)
Mid Aorta	417 (83.4%)
Distal Aorta	393 (78.6%)

**Table 3:** Means and standard deviations (age, weight) or numbers and percentages (male gender) for those with and those without visualization of the specified aortic segments * Based on logistic regression models + Adjusted for other predictors (Age, weight, and gender)

		Aorta Visualized Mean (SD) or N (%)		P-value for Difference*	
				Unadjusted	Adjusted^+^
Proximal Aorta		Yes (N=454)	No (N=46)		
	Age (Years)	66.9 (18.4)	68.0 (19.4)	0.72	0.35
	Weight (kg)	75.8 (19.5)	84.0 (21.8)	0.0098	0.0095
	Gender (Male)	237 (52.2%)	27 (58.7%)	0.40	0.79
Mid Aorta		Yes (N=417)	No (N=83)		
	Age (Years)	66.7 (18.4)	68.9 (18.9)	0.31	0.20
	Weight (kg)	76.1 (19.0)	79.0 (23.6)	0.22	0.16
	Gender (Male)	218 (52.3%)	46 (55.4%)	0.60	0.84
Distal Aorta		Yes (N=393)	No (N=107)		
	Age (Years)	66.4 (18.7)	69.4 (17.6)	0.13	0.052
	Weight (kg)	75.7 (19.1)	79.7 (22.2)	0.071	0.019
	Gender (Male)	209 (52.2%)	55 (51.4%)	0.74	0.40

## Discussion

In the ED, patients arrive in undifferentiated shock regularly. ED clinicians must maintain a high clinical suspicion for AAA in patients with abdominal pain and shock. The bedside ultrasound is a valuable tool because these unstable patients can be kept within arm’s reach of critical care resources rather than sending them to the radiology department. Although ultrasound image acquisition is operator dependent, even the most seasoned sonographer can be heard anecdotally questioning the feasibility of performing an abdominal aortic scan on a patient who is significantly overweight. The ultrasound relies on the propagation of soundwaves to produce a visual image. The further the image is from the probe, the more likely the sound waves will be scattered, producing an unreliable image, as is the case with an obese individual [21].

This study demonstrates that although there was a marginal difference in the ability to visualize the distal aorta in relation to weight (75.7 kg visualized versus 79.7 kg unvisualized), this difference of 4 kg is relatively small and likely to be clinically ambiguous. Furthermore, the standard deviation was 19.1 kg for the group where the distal aorta was visualized and 22.2 kg for the group where the distal aorta was not visualized. Studies have examined the accuracy of patient self-reported weight and noted that patients are not completely accurate in reporting their weight [22,23]. Therefore, given the overlap, weight should not be used to routinely exclude aortic scans.

In Table 1, the weight of included versus excluded ultrasounds had a p-value of 0.029, presenting potential for difference between populations included and excluded in this study. Despite the p-value, the mean weight difference between the two groups is less than 4kg, making the small weight difference seem unlikely to be clinically relevant. The excluded scans included those with incomplete documentation.

The most common reason for imaging inadequacy was the presence of bowel gas, and this did not correlate to weight. Although the distal aorta was not visualized in 21.4% of the patients included in this study, the corollary is that bedside ultrasound imaging was helpful in reducing immediate concern for AAA in 78.6% of patients. This enabled the ED clinician to re-evaluate their differential in real time during the acute resuscitation phase of the patient’s ED course. Thus, we recommend that a patient’s weight should not be a contraindication to performing bedside abdominal aortic scans.

Limitations

We acknowledge several limitations in this study. Our sample size was relatively small. While we were able to analyze 500 scans, nearly a third of the sample was excluded because of incomplete documentation. As it can be difficult to interpret upon later review what portion of the aorta was captured during the initial scan, studies without complete documentation were excluded. While most incomplete worksheets did not specifically comment on what limited their ability to visualize the aorta, factors other than aortic visualization could have contributed to their incomplete status. Given that the purpose of this study was to examine if a weight had any influence on the ability to visualize the aorta, the lack of sonographer reasoning is a large limitation. Clinicians of varying experience were included in this study, and although all the scans were performed under the supervision of a qualified sonographer and reviewed by an emergency ultrasound fellowship-trained physician, image acquisition is still operator dependent. A selection bias might also have been involved, given that there was skepticism about successfully obtaining an aortic scan on overweight individuals, leading a clinician to either not perform or not record an abdominal ultrasound. Finally, the use of two different ultrasound devices was a limitation because each device displays images differently, which may influence the clinician’s ability to acquire images.

## Conclusions

Bedside ultrasound is an important tool in helping to narrow down the differential and diagnose AAAs, especially in critically ill ED patients. In a typical ED, diversity exists among patients’ gender, body habitus, and other characteristics. In this study, patient weight, gender or age did not significantly clinically impact the ultrasound operators’ ability to visualize the distal aorta. Based on this study, a patient’s weight, gender, or age should not play a key role in the emergency clinician’s decision to scan the distal aorta on a bedside ultrasound.
